# A metagenomic study of the gut microbiome in patients with type 2 diabetes mellitus and myocardial infarction

**DOI:** 10.1007/s00592-026-02648-x

**Published:** 2026-02-09

**Authors:** Honghong Zhang, Changlin Zhai, Huilin Hu, Gang Qian, Menghui Mao

**Affiliations:** First Hospital of Jiaxing, No. 1882, Zhonghuan South Road, Nanhu District, Jiaxing City, Zhejiang Province China

**Keywords:** Gut microbiome, Type 2 diabetes mellitus, Myocardial infarction, Metagenomics

## Abstract

**Objective:**

This study aimed to investigate gut microbiota composition and metabolic functions in patients with type 2 diabetes mellitus (DM) complicated by myocardial infarction (MI) and to explore potential mechanisms linking the gut microbiome to MI development.

**Methods:**

Sixty patients with DM complicated by MI and 52 patients with DM alone were initially recruited. After quality control, 29 DM + MI patients and 33 DM patients were included in the final analysis. Gut microbial profiles were characterized using shotgun metagenomic sequencing and bioinformatics analyses. Microbial diversity, composition, and gene functions were compared between groups based on KEGG, COG, and CAZy annotations.

**Results:**

Overall microbial diversity and metabolic profiles were comparable between the two groups; however, significant differences were observed in specific taxa and functional genes. Taxa enriched in the DM + MI group included Bacteroidales, Prevotellaceae, and Lachnospiraceae. In total, 510 KEGG orthology (KO) units and 21 pathways—including ABC transporters, quorum sensing, and general metabolic pathways—differed significantly between groups. Carbohydrate transport and metabolism, as well as glycoside hydrolase activity, represented the most enriched functional categories. Random forest models based on selected microbial species,* KO units*, and KEGG pathways achieved areas under the curve (AUCs) of 0.868, 0.885, and 0.820, respectively.

**Conclusion:**

Patients with DM complicated by MI exhibit distinct gut microbial compositions and functional gene signatures compared with patients with DM alone. These microbiome-based markers may contribute to early risk stratification and provide potential targets for microbiota-focused interventions to mitigate MI risk in patients with diabetes.

**Supplementary Information:**

The online version contains supplementary material available at 10.1007/s00592-026-02648-x.

## Introduction

Atherosclerotic cardiovascular disease (ASCVD) remains the leading cause of death among patients with DM, who frequently present with extensive multivessel coronary artery disease and poor long-term outcomes. Although treatment strategies for MI are broadly similar across populations, patients with DM have a higher incidence of complications, including heart failure, arrhythmias, renal dysfunction, and recurrent infarction.

Globally, the prevalence of diabetes increased from 108 million in 1980 to 422 million in 2014, with approximately 5% of the population remaining undiagnosed. Although advances in therapy have contributed to a reduction in MI incidence, the growing prevalence of DM has offset much of this benefit [1–3].

Diabetes is an established independent risk factor for ASCVD, with cardiovascular risk rising in parallel with glycemic burden. For STEMI, immediate percutaneous coronary intervention (PCI) is recommended, whereas for NSTEMI, an early invasive strategy is preferred. Regardless of presentation, intensive secondary prevention—including aspirin, high-intensity statins, β-blockers, renin–angiotensin–aldosterone system (RAAS) inhibitors, sodium–glucose cotransporter 2 (SGLT2) inhibitors, and glucagon-like peptide-1 (GLP-1) receptor agonists—is required.

The disproportionate cardiovascular burden among individuals with DM presents a major public health and socioeconomic challenge, underscoring the urgent need to identify novel risk factors and therapeutic strategies. However, the mechanisms underlying the poor prognosis of MI in patients with DM remain incompletely understood.

Emerging evidence suggests that the gut microbiota may play a pivotal role in this relationship through metabolic disruption and systemic inflammation. Patients with both DM and acute MI exhibit distinct alterations in gut microbial composition—characterized by an increase in Firmicutes and a decrease in Bacteroidetes—compared with non-diabetic MI patients, indicating dysbiosis that may contribute to disease progression and adverse cardiovascular outcomes [4]. Moreover, gut microbial imbalance has been implicated in a broad spectrum of DM-related complications, including ASCVD, thereby highlighting the gut microbiome as a potential therapeutic target in cardiometabolic disorders [5].

Several studies have examined the potential association between gut microbiota and MI in DM, focusing on mechanisms such as inflammatory signaling and microbial metabolic modulation. However, these findings may have been confounded by heterogeneity in clinical characteristics, medication use, and environmental factors, and definitive conclusions remain elusive.

Therefore, in this study, we applied strict inclusion and exclusion criteria to minimize confounding factors and performed metagenomic sequencing to comprehensively characterize the gut microbiota composition in patients with DM complicated by MI compared with those with DM alone.

## Methods

### Participants

Patients were enrolled according to the ***Fourth Universal Definition of Myocardial Infarction (2018)*** [6] and the ***Standards of Medical Care in Diabetes—2022*** [7]. Exclusion criteria included: (1) diarrhea, inflammatory bowel disease, or other infectious conditions; (2) autoimmune disorders or malignancies; (3) recent chemotherapy, radiotherapy, or surgery; (4) use of antibiotics, corticosteroids, acid suppressants, herbal medicines, probiotics (e.g., yogurt), or dietary supplements within the previous 3 months; (5) other organic gastrointestinal diseases; (6) history of gastrointestinal surgery; and (7) menstruation at the time of stool collection. Fresh stool samples were collected, stored at − 80 °C, and subjected to metagenomic sequencing. The study was approved by the Ethics Committee of the First Affiliated Hospital of Jiaxing University, and written informed consent was obtained from all participants (No. 2022-LY-226). This study was registered in the Chinese Clinical Trial Registry (ChiCTR2200063341).

## Metagenomic sequencing

Microbial DNA was extracted from fecal samples using the DNA Stool Kit (APPLYGEN, Shanghai, China) according to the manufacturer’s instructions. DNA integrity was assessed by 1% agarose gel electrophoresis. Genomic DNA was fragmented to ~ 300 bp using a Covaris M220 system, and paired-end libraries were prepared with the TruSeq™ DNA Sample Prep Kit. Cluster generation was performed using the HiSeq 3000/4000 PE Cluster Kit through bridge amplification, followed by sequencing on the Illumina HiSeq 3000/4000 platform with the SBS Kit, which utilizes fluorescently labeled nucleotides and laser-based detection for base incorporation.

Raw reads were processed with **fastp** (v0.19.5) to remove adapters, low-quality bases, ambiguous bases (N), and short reads, yielding high-quality clean reads. De novo assembly was performed with **MEGAHIT** using an iterative k-mer strategy. Open reading frames (ORFs) were predicted with **MetaGene** [8], and genes ≥ 100 bp were translated into amino acid sequences.

A non-redundant gene catalog was constructed with **CD-HIT** [9] at 95% identity and 90% coverage, retaining the longest sequence as the representative. Taxonomic and functional annotation was conducted using **DIAMOND** (v2.0.13) in BLASTP mode (e-value < 1e − 5) against multiple databases, including NR (NCBI non-redundant protein database), KEGG [10], EggNOG, and CAZy [11]. Gene and functional abundances were quantified for comparative analyses.

## Bioinformatic analysis

Venn diagrams were constructed to illustrate shared and unique species, functions, or genes among groups. Heatmaps were generated using the **vegan** *package in R to cluster samples at the species*,* COG*,* and KEGG levels*, thereby visualizing compositional similarities and differences.

Community diversity and structure were assessed by principal component analysis (PCA), principal coordinate analysis (PCoA), non-metric multidimensional scaling (NMDS), and analysis of similarities (ANOSIM), performed with **QIIME2** (version 2021.4) [12].

Between-group taxonomic differences were evaluated using linear discriminant analysis effect size (LEfSe) [13] and the Wilcoxon rank-sum test. LEfSe analyses were conducted via the LEfSe platform [14]. LEfSe analyses used α = 0.05 for the Kruskal–Wallis test and an LDA score cutoff of 2.0 (default), unless otherwise specified.

Random forest models were used to identify discriminative microbial markers and to assess predictive capacity between the DM and DM + MI groups. Feature importance was quantified using the Mean Decrease Accuracy (MeanDecreaseAccuracy) metric, which measures the decrease in classification accuracy when the values of a given feature are randomly permuted, thereby reflecting its contribution to model performance. Microbial species were ranked according to their importance scores, and the top-ranked features were sequentially included to construct classification models. Model performance was evaluated using 10-fold cross-validation and quantified by receiver operating characteristic (ROC) curves and the corresponding AUC. The optimal number of features was determined based on the highest AUC achieved across models.

### Statistical analysis

Statistical analyses were conducted using R (v4.3.2). Continuous variables were summarized as medians or means ± standard deviations, and group differences were tested using the *t*-test or Wilcoxon rank-sum test, as appropriate. Categorical variables were expressed as counts and percentages, with comparisons performed using the chi-square test or Fisher’s exact test. A two-sided α level of 0.05 was considered statistically significant. After false discovery rate (FDR) correction, *p* < 0.01 was applied as the threshold for significance.

## Results

### Clinical characteristics of the participants

**Fig. 1:**
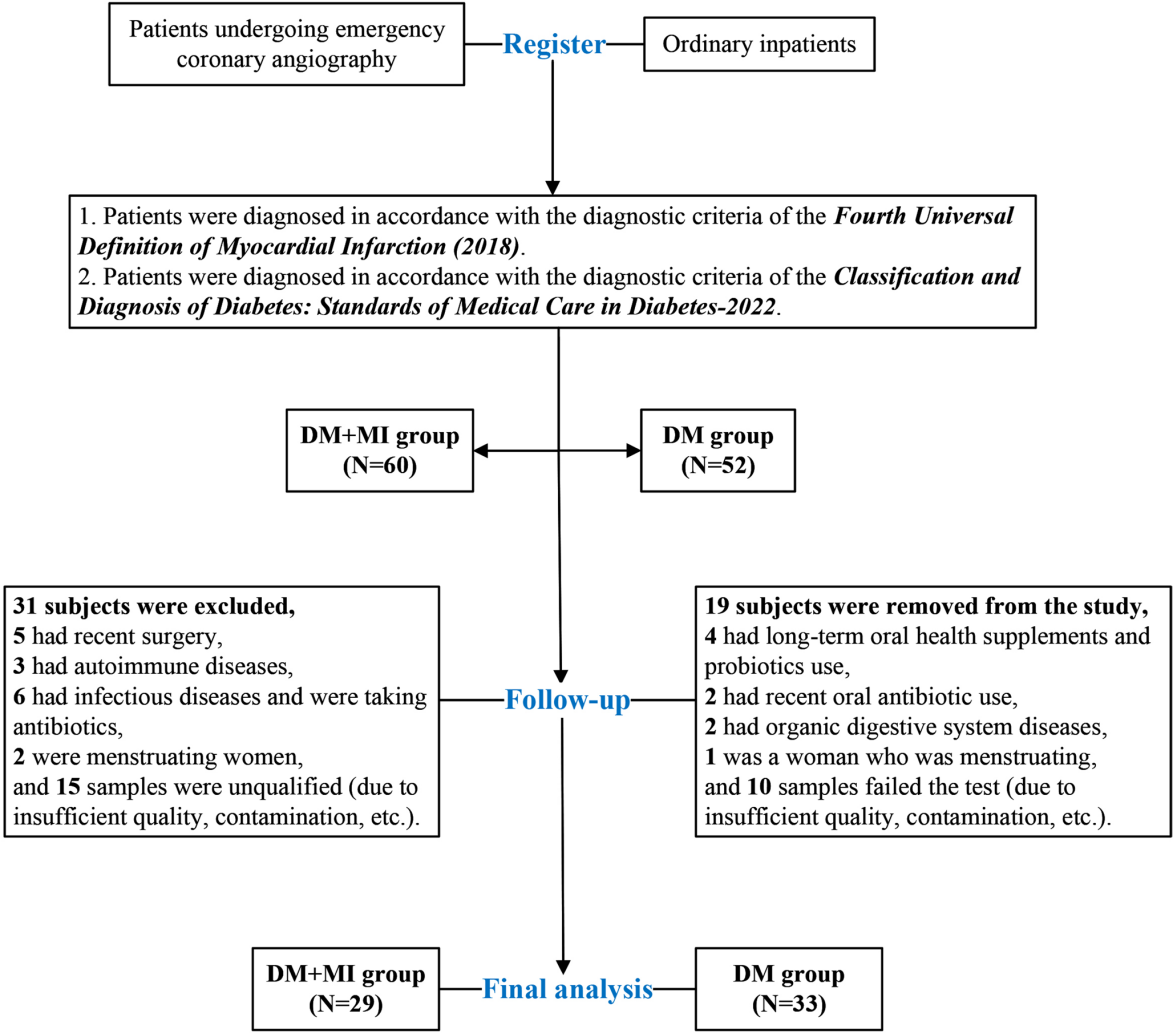
Flowchart of patient enrollment and exclusion

A total of 112 patients were initially screened for eligibility. Of these, 50 were excluded due to recent surgery, autoimmune or infectious diseases, antibiotic or probiotic use, menstruation, or inadequate/contaminated stool samples. Ultimately, 62 patients were included in the final analysis, comprising 29 in the DM + MI group and 33 in the DM group (Fig. 1). Baseline clinical characteristics of the study population are presented in Table 1.

## Species composition and abundance analysis

### Gut Microbiome diversity

**Fig. 2:**
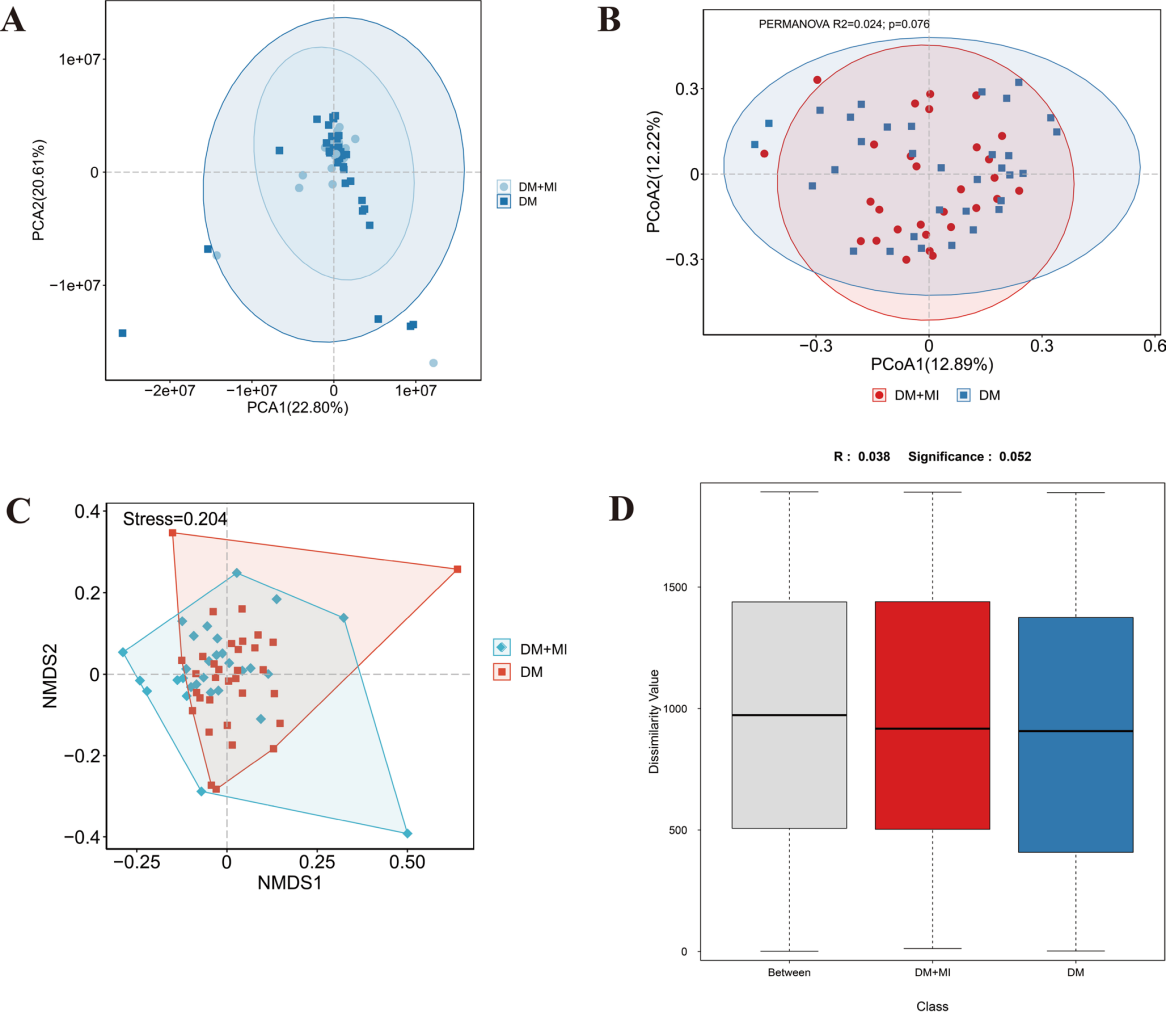
β-diversity of the gut microbiota between DM + MI and DM groups. (A) Principal component analysis (PCA) at the species level, showing that the first two principal components explained 22.80% and 20.61% of the total variance, respectively. (B) Principal coordinates analysis (PCoA) based on the Bray–Curtis dissimilarity matrix, with the first two axes accounting for 12.89% and 12.22% of the variation; the two groups showed substantial overlap without distinct clustering. (C) Non-metric multidimensional scaling (NMDS) ordination derived from Bray–Curtis dissimilarities, with a stress value of 0.204 indicating an acceptable two-dimensional representation. (D) Results of permutation-based statistical tests for between-group differences. PERMANOVA did not detect significant dissimilarity (R² = 0.024, *p* = 0.076), and ANOSIM similarly indicated no significant group separation (*R* = 0.038, *p* = 0.052)

The gut microbiome exhibits complex and dynamic diversity that influences host metabolism, immune regulation, and disease susceptibility. In this study, we focused on β-diversity to evaluate community-level differences between the DM + MI and DM groups at the species level. Variance-based PCA and distance-based ordination methods (PCoA and NMDS) were performed using the Bray–Curtis dissimilarity matrix, and statistical significance was assessed with permutation-based approaches (PERMANOVA and ANOSIM) (Fig. 2A–D). In the PCA plot, the first and second principal components explained 22.80% and 20.61% of the total variance, respectively (Fig. 2A). The PCoA ordination accounted for 12.89% and 12.22% of the variation, showing substantial overlap between groups and no apparent clustering (Fig. 2B). Consistently, PERMANOVA (R² = 0.024, *p* = 0.076) did not identify a significant difference in overall community composition. NMDS analysis yielded a stress value of 0.204, indicating a moderately reliable two-dimensional representation. Although a partial separation was visually apparent, ANOSIM (*R* = 0.038, *p* = 0.052) confirmed the absence of significant between-group dissimilarity (Fig. 2C–D). Taken together, these complementary analyses indicate that the gut microbiota compositions of the DM + MI and DM groups were broadly comparable, although a subtle trend toward separation could not be excluded.

### Comparative analysis of gut microbiota composition

**Fig. 3:**
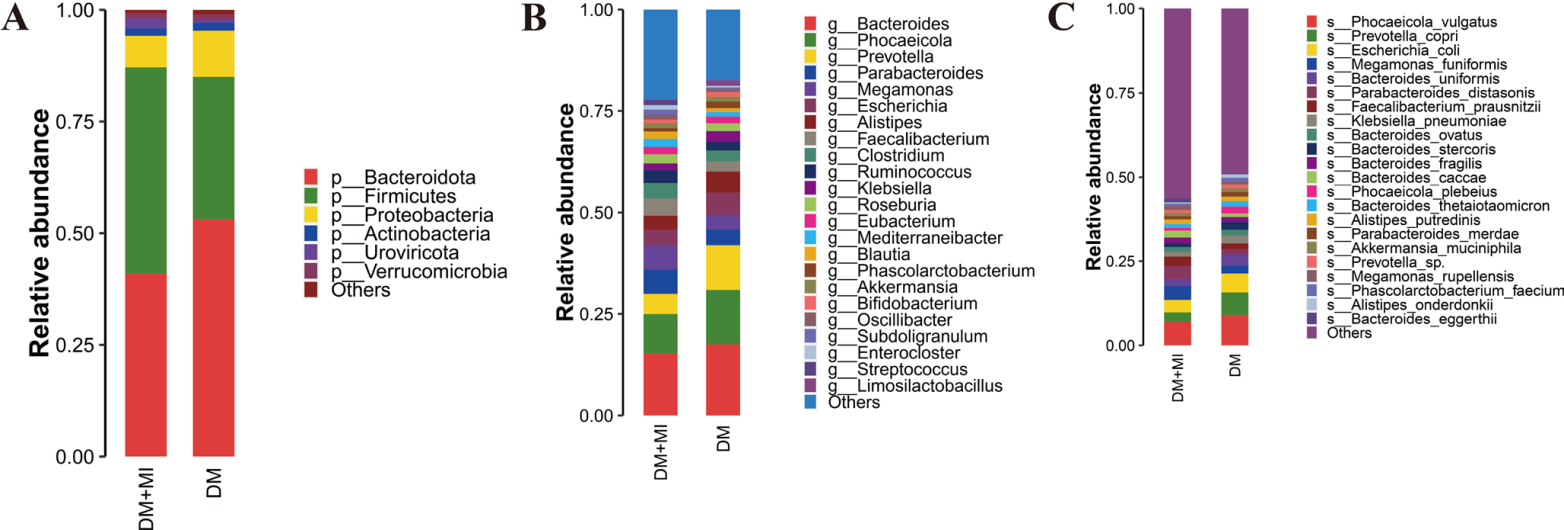
Stacked bar plots of the gut microbiota composition in the DM + MI and DM groups. (A) Phylum level. (B) Genus level. (C) Species level

Metagenomic sequencing revealed the relative abundances of microbial taxa across multiple taxonomic levels, which were aggregated to construct community profiles. Stacked bar plots were generated at the phylum, genus, and species levels to visualize the distribution of dominant taxa in each group (Fig. 3A–C), with major high-abundance taxa labeled accordingly.

**Table 1:** **Population characteristics**

*Variables*	*DM* (*N* = 33)	*DM + MI* (*N* = 29)	*P-value*
*Age (year)*	*59 (49*,* 64)*	*64 (56*,* 69)*	*1.48E-01*
*BMI (kg/m* ^*2*^ *)*	*23.63 ± 2.76*	*25.11 ± 3.00*	*4.83E-02*
*Male*	*16 (48.48%)*	*24 (82.76%)*	*7.44E-03*
*Smoke*	*6 (18.18%)*	*15 (51.72%)*	*2.40E-02*
*Drink*	*8 (24.24%)*	*6 (20.69%)*	*9.77E-01*
*Hypertension*	*21 (63.64%)*	*19 (65.52%)*	*1.00E + 00*
*Hyperlipidemia*	*9 (27.27%)*	*10 (34.48%)*	*7.35E-01*
*HbA1c [% (mmol/mol)]*	*10.80 (8.20*,* 12.00)*	*6.60 (6.50*,* 7.30)*	*1.43E-08*
**Hypoglycemic drugs**			
*Metformin*	*22 (66.67%)*	*5 (17.24%)*	*2.53E-04*
*GLP-1 receptor agonists*	*2 (6.06%)*	*1 (3.45%)*	*1.00E + 00*
*SGLT2 inhibitors*	*9 (27.27%)*	*3 (10.34%)*	*1.16E-01*
*Insulin*	*28 (84.85%)*	*5 (17.24%)*	*4.01E-07*

### Data are presented as absolute %, mean ± SD, or median (first quartile, third quartile)

**Fig. 4:**
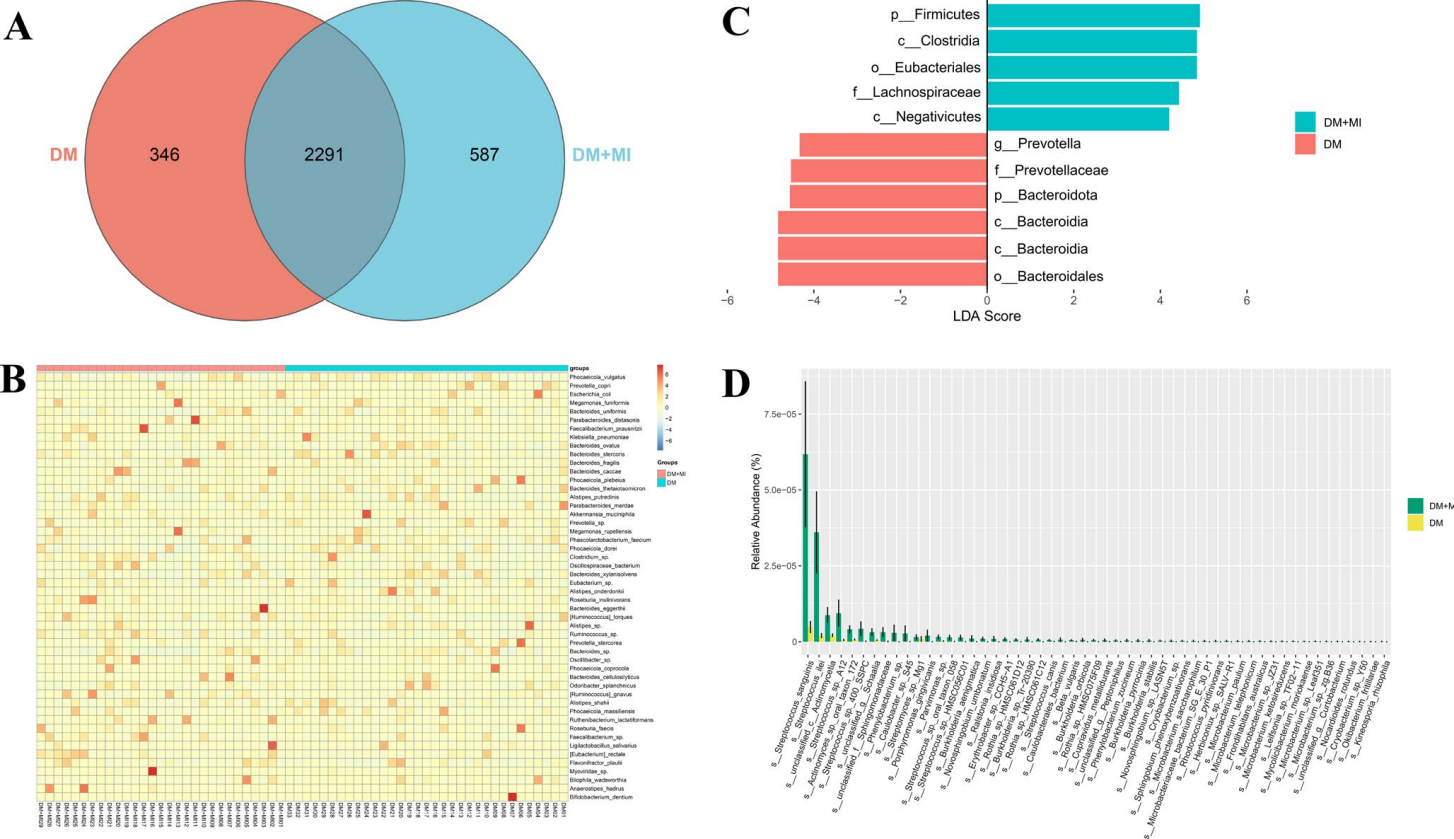
Differential taxonomic analysis of the gut microbiota between groups. (A) Venn diagram showing shared and unique species in each group. (B) Heatmap of the top 50 most abundant species across samples. (C) LEfSe analysis identifying group-discriminative taxa at multiple taxonomic levels, presented by LDA scores. (D) Wilcoxon rank-sum test with FDR correction displaying species ranked by relative abundance differences between groups

At the species level, presence–absence profiles were compared between groups. The Venn diagram showed that the DM group contained 2,637 species and the DM + MI group 2,878 species, of which 2,291 were shared; 346 species were unique to the DM group and 587 to the DM + MI group (Fig. 4A).

To visualize relative abundance patterns across individuals, a heatmap of the top 50 species was generated, revealing compositional heterogeneity among participants but overall similarity within groups (Fig. 4B).

Differential taxa were further identified using LEfSe, which integrates non-parametric testing with linear discriminant analysis to detect class-specific biomarkers. LEfSe highlighted discriminative features across multiple taxonomic ranks (Fig. 4C), with Bacteroidota/Bacteroidia/Bacteroidales, Prevotellaceae, *Prevotella*, and *P. copri* enriched in one group. In contrast, Firmicutes/Clostridia/Eubacteriales, Lachnospiraceae, and Negativicutes were enriched in the other (Fig. 4C).

Feature-level testing was also conducted using the Wilcoxon rank-sum test with Benjamini–Hochberg correction (q < 0.01). Among 2,023 species tested, 938 remained significant after FDR adjustment. Species were ranked by effect size, with *Streptococcus sanguinis* showing the largest between-group difference, followed by *Streptococcus ilei*, unclassified *Actinomycetia*, and *Streptococcus* sp. A12 (Fig. 4D).

### Diagnostic model based on microbial species

**Fig. 5:**
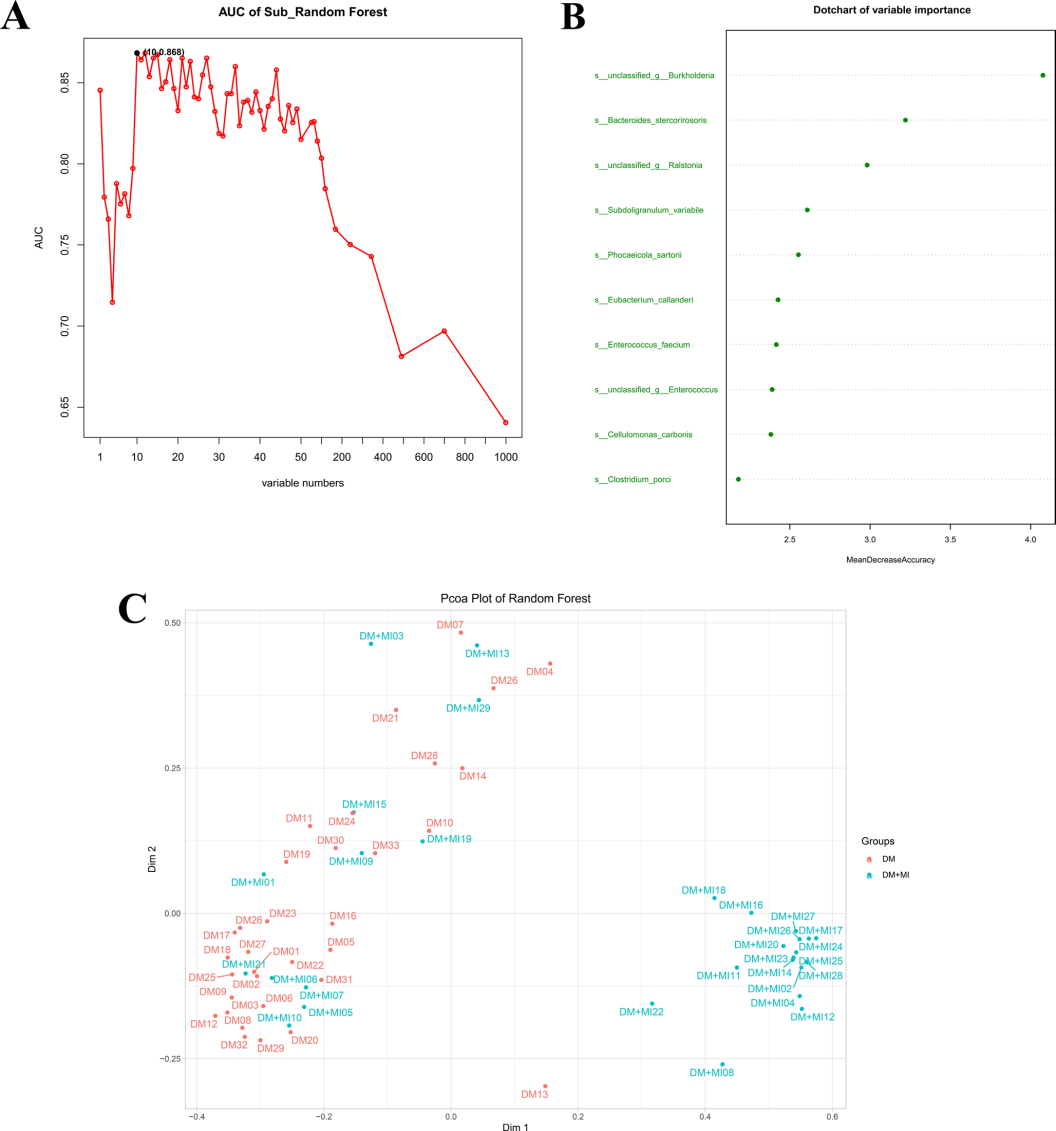
Random-forest–based prediction using species-level gut microbiota. (A) AUC as a function of the number of top-ranked species; peaks at 10 features (AUC = 0.868). (B) Dotchart of variable importance (MeanDecreaseAccuracy) for the top 10 species. (C) PCoA based on the random-forest proximity matrix, showing partial separation between groups

A random forest model with 10-fold cross-validation was constructed at the species level to evaluate and predict MI risk in patients with DM. Model performance was assessed using ROC analysis. As shown in Fig. 5A, the best classification accuracy was achieved when the top 10 most important species were included, yielding an AUC of 0.868. Variable importance ranking (MeanDecreaseAccuracy) highlighted the key discriminative species used by the model (Fig. 5B*)*: *unclassified Burkholderia*, *Bacteroides stercoris*, *unclassified* *Ralstonia*, *Subdoligranulum variabile*, *Phocaeicola sartorii*, *Eubacterium callanderi*, *Enterococcus faecium*, *unclassified* *Enterococcus*, *Cellulomonas carbonis*, and *Clostridium porci*. A PCoA of the random forest proximity matrix (Fig. 5C) revealed partial separation between the DM and DM + MI groups along the first coordinate, although some overlap remained.

### Functional level analysis

**Fig. 6:**
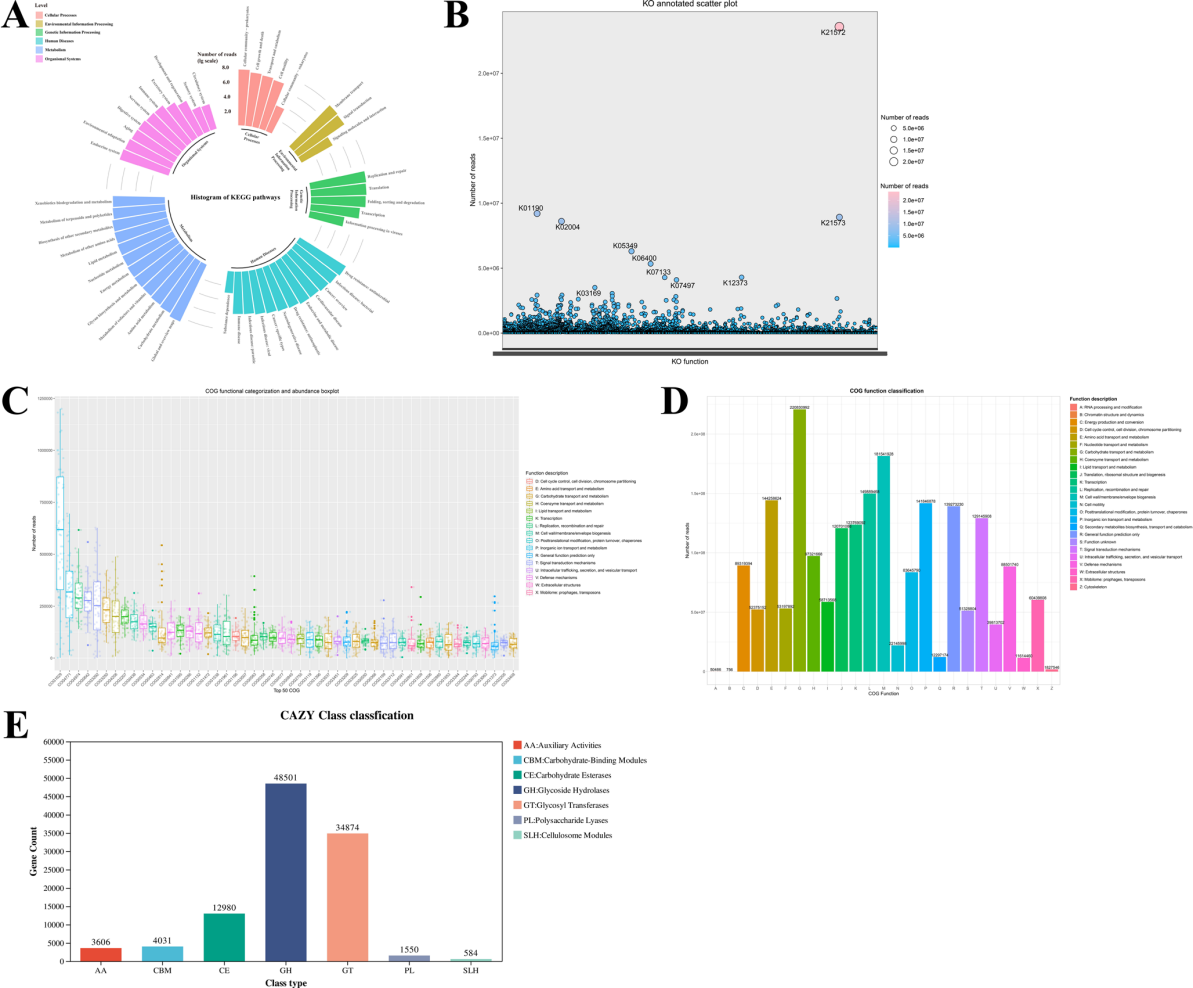
Functional profiling of the gut microbiome based on multi-database annotation. (A) KEGG pathway abundances at level-1 and level-2; (B) Abundances of the top KOs; (C) Heatmap of the top 50 abundant COGs; (D) Classification of abundant COGs by functional group; (E) CAZy enzyme class annotation and abundance

*Functional annotation of the nonredundant gene catalog was conducted using KEGG*,* COG*,* and CAZy databases. KEGG mapping identified 6 level-1 categories*,* 47 level-2 pathways*,* and 415 level-3 pathways*,* with metabolism being the most enriched—particularly global and overview maps and carbohydrate metabolism. Environmental information processing (mainly membrane transport) and genetic information processing ranked second and third*,* respectively (*Fig. 6A*). In total*,* 7*,*742 KOs were detected*,* several of which exhibited high relative abundances (*Fig. 6B*).*

COG annotation assigned 4,460 orthologous groups across 25 functional classes within four major categories. The 50 most abundant COGs are shown in Fig. 6C, with their class-level distribution summarized in Fig. 6D.

CAZy annotation revealed 7 enzyme classes encompassing 545 families, dominated by glycoside hydrolases (GHs) and glycosyl transferases (GTs) (Fig. 6E).

To assess functional diversity, PCA, PCoA, and NMDS analyses were conducted based on KEGG KO and pathway profiles. Consistent with the taxonomic diversity results, KO-level analysis demonstrated modest but statistically significant differences between the DM and DM + MI groups (ANOSIM, *R* = 0.060, *p* = 0.010), whereas pathway-level analysis showed no significant separation (ANOSIM, *R* = 0.014, *p* = 0.189) (**Supplementary Fig. 1**).

Functional composition at the KO and pathway levels was compared between the DM and DM + MI groups using KEGG-based annotation. Venn analysis revealed a large shared core alongside group-specific KOs and pathways, while heatmap visualization highlighted distinct distribution patterns across samples. LEfSe analysis identified 66 KOs and 19 pathways with significantly different relative abundances,* including K21572*,* K06400*, and pathways such as ABC transporters and metabolic pathways. These discriminative features, supported by LDA scores above the significance threshold, may represent potential functional biomarkers. Wilcoxon rank sum testing with multiple comparison correction further validated several differential KOs and pathways (**Supplementary Figs. 2–3**).

Random forest models were further applied to KEGG KO units and pathways. Optimal performance was achieved when the top 49 KO units and top 7 pathways were included, yielding AUCs of 0.885 and 0.820, respectively. PCoA based on the model proximity matrix revealed partial separation between the DM and DM + MI groups, while variable importance plots highlighted the most discriminative KO units and pathways (**Supplementary Fig. 4**).

## Discussion

This study employed metagenomic sequencing to characterize the gut microbiota of DM patients with and without MI, focusing on taxonomic composition, functional profiles, and metabolic pathways. The objectives were to identify microbial species associated with MI in DM and to explore functional and metabolic alterations underlying these changes, thereby providing potential biomarkers for early diagnosis and microbiota-targeted interventions. The main findings were: (1) although overall species and pathway diversity did not differ markedly, functional gene structures and the relative abundances of several taxa and pathways varied significantly between groups; (2) differentially abundant taxa included *Bacteroidales*, *Bacteroidia*, *Bacteroidota*, *Prevotellaceae*, *Prevotella*, *P. copri*, *Negativicutes*, *Lachnospiraceae*, *Eubacteriales*, *Clostridia*, and *Firmicutes*, *along with 21 KEGG level-3 pathways (e.g.*,* ABC transporters*, metabolic pathways,* quorum sensing)* and 510 KO units, all of which may contribute to MI pathogenesis; and (3) a random forest model integrating the top 10 microbial species, 49 KO units, and 7 KEGG pathways achieved high predictive accuracy, highlighting their potential as discriminative biomarkers of MI risk in DM patients.

Initial analyses showed no significant differences in overall gut microbial diversity between groups; however, compositional separation was evident, suggesting dysbiosis in DM + MI patients relative to DM. Under physiological conditions, the gut microbiota maintains a dynamic equilibrium that supports host metabolism, immune regulation, and pathogen defense. In DM + MI patients, this balance appeared disrupted, indicating reduced ecosystem stability and insufficient suppression of potentially harmful taxa. Such dysbiosis highlights the importance of microbial homeostasis and provides a foundation for understanding disease-associated microbial alterations [15, 16]. Previous studies have consistently linked gut microbial imbalance with both DM and MI, and the present metagenomic analysis offers new insights into microbial mechanisms underlying MI risk in DM.

Taxonomic profiling revealed that both groups were dominated by *Bacteroidota*, *Firmicutes*, *Proteobacteria*, *Actinobacteria*, *Uroviricota*, and *Verrucomicrobia* at the phylum level; *Bacteroides*, *Phocaeicola*, *Prevotella*, *Parabacteroides*, *Megamonas*, and *Escherichia* at the genus level; and *P. vulgatus*, *P. copri*, *E. coli*, *M. funiformis*, *B. uniformis*, and *P. distasonis* at the species level. A comparative analysis revealed significant enrichment of *Firmicutes*, *Clostridia*, *Eubacteriales*, *Lachnospiraceae*, and *Negativicutes* in DM + MI patients, as identified by LEfSe. Among the most discriminative taxa were *Streptococcus sanguinis*, *Streptococcus ilei*, and unclassified *Actinomycetia*. *S. sanguinis* is known to possess virulence factors such as biofilm formation, platelet aggregation, and endothelial adhesion, and can modulate host immunity via oxidative stress and neutrophil extracellular traps, thereby contributing to cardiovascular pathology [17–20]. Although evidence remains limited, *S. ilei* has also been implicated in pathogenic processes [21], while *Actinomycetia* members are commonly associated with infections [22]. These findings suggest that specific taxa may be mechanistically linked to MI development in DM, warranting further investigation.

Functional annotation across multiple databases revealed potential microbial mechanisms contributing to disease progression. EggNOG analysis showed enrichment of COG categories related to carbohydrate transport and metabolism [G], cell wall/membrane/envelope biogenesis [M], and transcription [K]. CAZy annotation highlighted glycoside hydrolases (GHs), glycosyl transferases (GTs), and carbohydrate esterases (CEs), consistent with the central role of gut microbes in carbohydrate utilization [23] and the importance of glycosylation for microbial stability and host interactions [24, 25]. *KEGG profiling emphasized metabolism as the dominant level-1 category*,* with global and overview maps*,* carbohydrate metabolism*,* and amino acid metabolism enriched at level-2. At the level-3*,* ABC transporters*,* quorum sensing*,* and the pentose phosphate pathway were significantly upregulated in DM + MI patients*,* all of which are closely linked to cardiovascular disease via cholesterol homeostasis*,* vascular function*,* inflammation*,* and platelet activation* [26–32]. Functional analysis also identified abundant SusD family proteins (K21572) involved in starch binding [33, 34], while discriminatory KOs, such as K06400 (spoIVCA), K03205 (virD4), and K07720 (yesN), were enriched in DM + MI. Collectively, these results demonstrate significant functional dysbiosis in DM + MI patients, consistent with previous reports [35–37], and highlight potential microbiota-based therapeutic strategies, such as dietary modulation, prebiotics, and probiotics [38].

Building on these results, a random forest model integrating the ten most discriminative microbial species, 49 KO units, and seven KEGG pathways distinguished DM + MI from DM patients with high accuracy. From a translational perspective, predicting MI risk based on gut microbiota taxa, functional units, and metabolic pathways remains relatively underexplored. The present findings suggest that in DM + MI patients, the gut microbiota may contribute to MI pathogenesis through multiple mechanisms, providing a novel framework for early diagnosis and targeted interventions.

 Several limitations should be acknowledged. First, this study was designed as an exploratory metagenomic investigation aimed at characterizing differences in gut microbial composition and function between patients with type 2 diabetes mellitus and those with diabetes complicated by myocardial infarction, rather than to establish causal relationships. As in other microbiome studies conducted in acute cardiovascular settings, baseline differences in demographic and clinical characteristics—including sex distribution, smoking status, glycemic control, and antidiabetic treatment—reflect real-world clinical heterogeneity and may have influenced the observed microbial profiles [39]. Therefore, residual confounding cannot be fully excluded, and the findings should be interpreted as associations rather than evidence of causality. Second, lifestyle factors such as smoking and alcohol consumption were recorded in a binary manner at enrollment, and more detailed classifications (e.g., current, former, or never use) were not uniformly available due to the retrospective and exploratory nature of the study. This limitation may have constrained a more granular assessment of lifestyle-related confounding and has been acknowledged accordingly. In addition, differential abundance was assessed using the Wilcoxon rank-sum test with multiple-testing correction; however, we acknowledge that more compositionally aware approaches (e.g., *ALDEx2*) may be better suited for microbiome data and were not applied in the present analysis. This should be considered a limitation, and future studies with larger sample sizes will incorporate such methods to further validate and extend our findings. Third, all participants were recruited from a single center within a single geographic region, which may reduce but does not eliminate potential confounding related to socioeconomic and regional factors. In addition, not all diabetes-related clinical information—such as disease duration and microvascular complications—was uniformly available for all participants. Mechanistic investigations were beyond the scope of the present study, further limiting direct biological inference. Moreover,the relatively small sample size may affect the generalizability of the findings. Future studies incorporating larger, multicenter cohorts, longitudinal sampling, more comprehensive clinical characterization, and experimental or integrative computational approaches are warranted to validate and extend these results.

## Conclusion

In conclusion, this study demonstrates distinct taxonomic and functional alterations in the gut microbiota of DM + MI patients compared with DM alone, highlighting potential microbial mechanisms driving MI risk in diabetes. These findings provide novel biomarkers that may inform future strategies for early diagnosis and microbiota-targeted interventions.

## Supplementary Information

Below is the link to the electronic supplementary material.

Supplementary Material 1

Supplementary Material 2

Supplementary Material 3

Supplementary Material 4

Supplementary Material 5

Supplementary Material 6
