# Supplementary figures and images for: A metagenomic study of the gut microbiome in patients with type 2 diabetes mellitus and myocardial infarction
Source: Acta Diabetol. 2026 Feb 9;63(5):789–99. doi: 10.1007/s00592-026-02648-x (PMC13219173; doi:10.1007/s00592-026-02648-x)

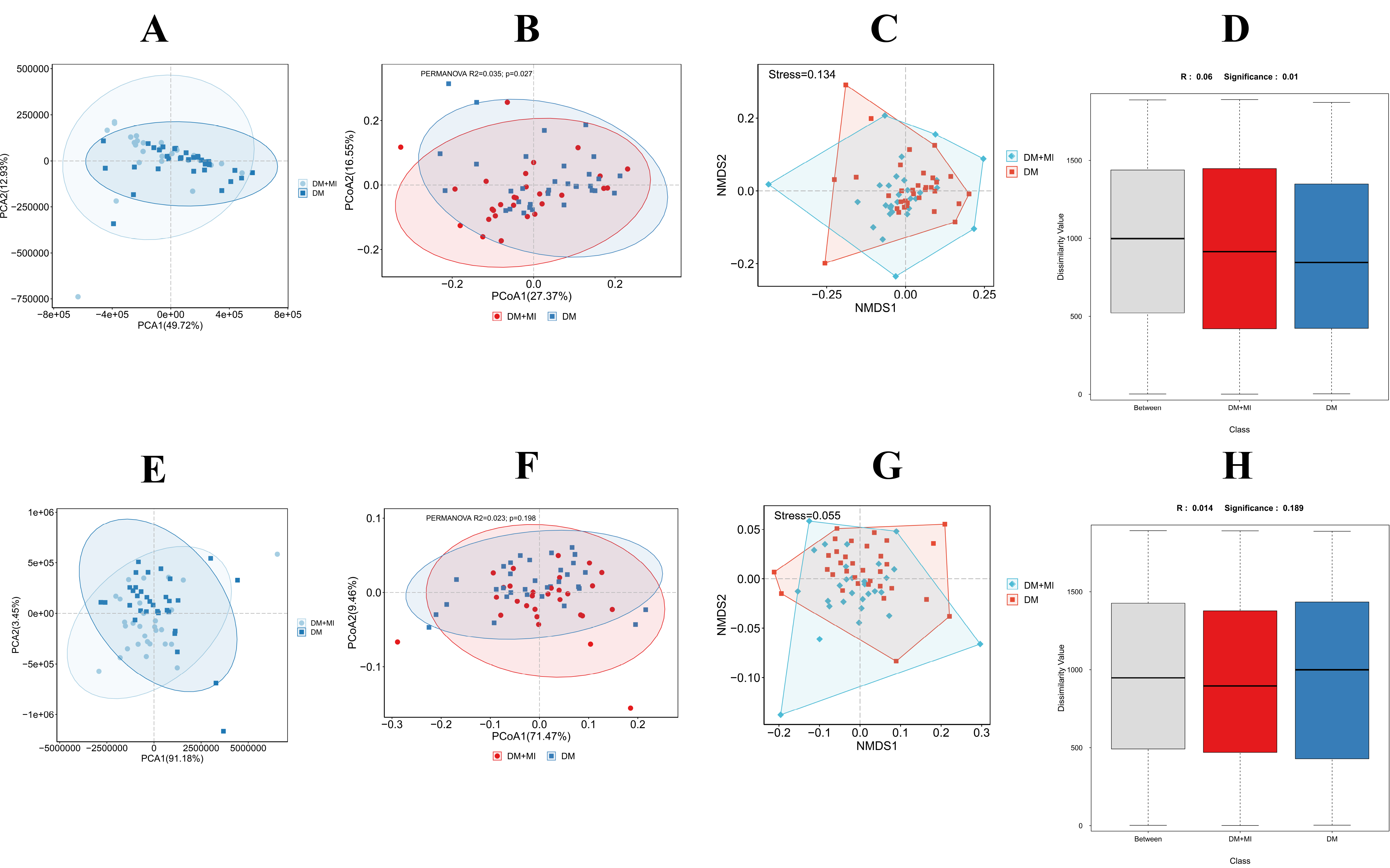

Supplement: Supplementary file 1 — Supplementary Material 1 [file 592_2026_2648_MOESM1_ESM.jpg]

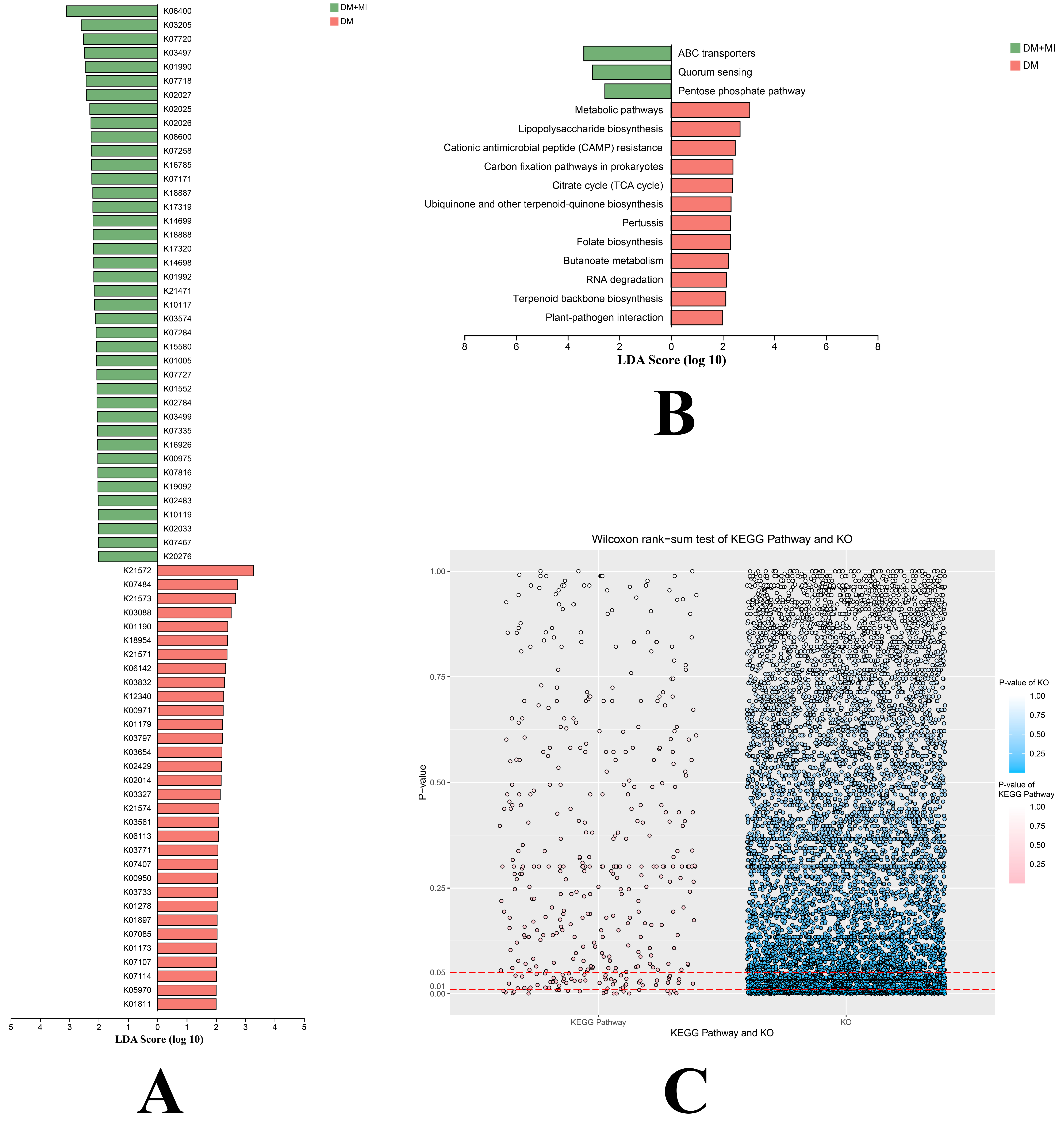

Supplement: Supplementary file 3 — Supplementary Material 3 [file 592_2026_2648_MOESM3_ESM.jpg]

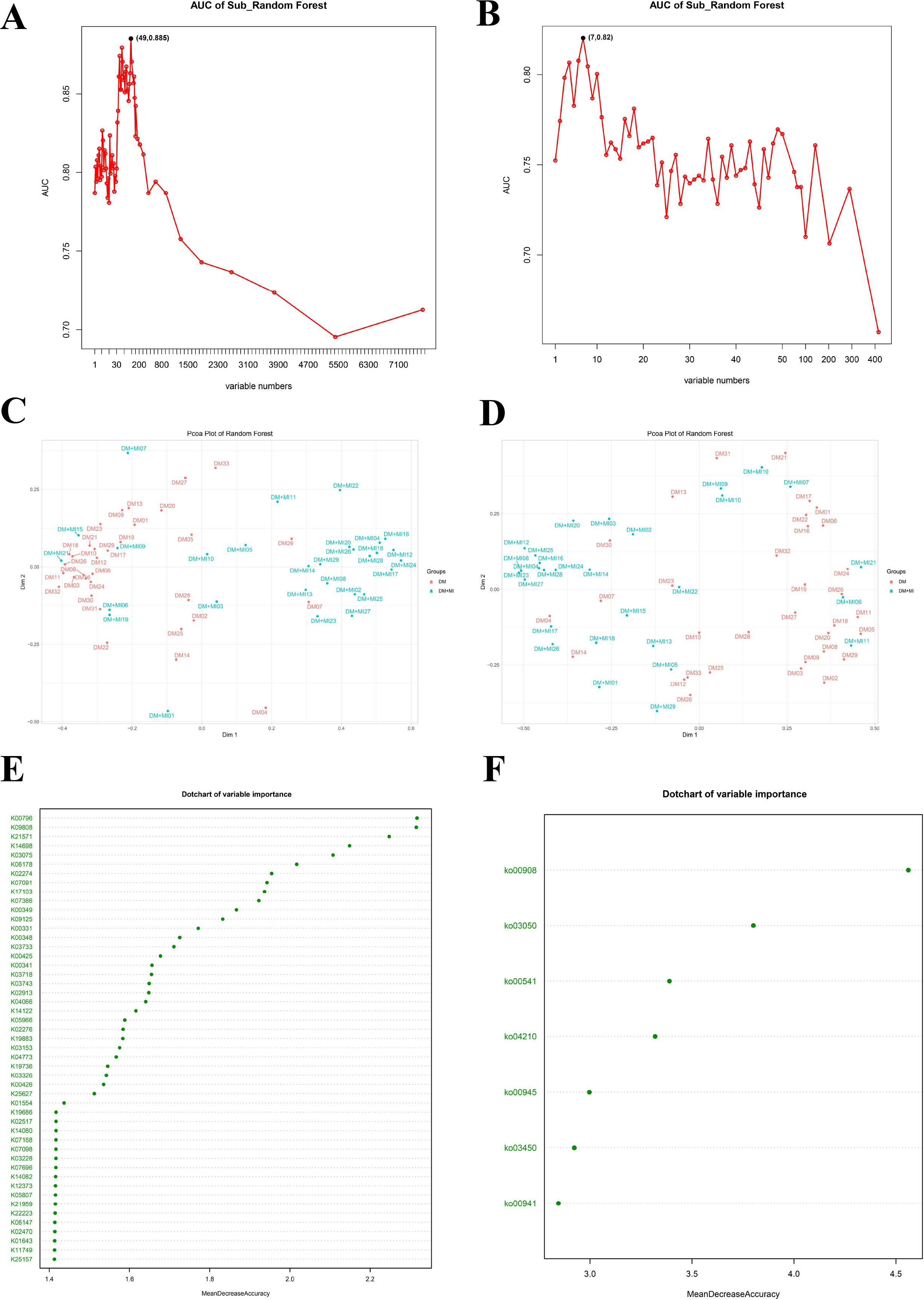

Supplement: Supplementary file 4 — Supplementary Material 4 [file 592_2026_2648_MOESM4_ESM.jpg]
