# Supplementary material for: A metagenomic study of the gut microbiome in patients with type 2 diabetes mellitus and myocardial infarction
Source: Acta Diabetol. 2026 Feb 9;63(5):789–99. doi: 10.1007/s00592-026-02648-x (PMC13219173; doi:10.1007/s00592-026-02648-x)
Supplement: Supplementary file 5 — Supplementary Material 5 [file 592_2026_2648_MOESM5_ESM.docx]

### Supplementary Figure Legends

**Supplementary Figure 1 | Functional diversity analyses based on KEGG KO and pathway profiles.**
 (A) Principal component analysis (PCA) based on KEGG ortholog (KO) functional units.
 (B) Principal coordinate analysis (PCoA) of KO functional units with PERMANOVA results.
 (C) Non-metric multidimensional scaling (NMDS) of KO functional units.
 (D) Analysis of similarities (ANOSIM) based on KO functional units.
 (E) PCA based on KEGG pathway functional units.
 (F) PCoA of KEGG pathway functional units with PERMANOVA results.
 (G) NMDS of KEGG pathway functional units.
 (H) ANOSIM based on KEGG pathway functional units.

**Supplementary Figure 2 | Venn diagrams and heatmaps of KEGG KO units and pathways.**
 (A) Venn diagram illustrating shared and unique KEGG KO functional units between groups.
 (B) Heatmap showing the relative abundance of dominant KEGG KO functional units across samples.
 (C) Venn diagram illustrating shared and unique KEGG pathways between groups.
 (D) Heatmap showing the relative abundance of dominant KEGG pathways across samples.

**Supplementary Figure 3 | Differential functional features identified by LEfSe and Wilcoxon analyses.**
 (A) Linear discriminant analysis effect size (LEfSe) of KEGG KO functional units with corresponding LDA scores.
 (B) LEfSe analysis of KEGG pathways with corresponding LDA scores.
 (C) Wilcoxon rank-sum test identifying significantly different KEGG KO units and pathways between groups.

**Supplementary Figure 4 | Random forest prediction models based on KEGG KO units and pathways.**
 (A) Receiver operating characteristic (ROC) curves showing the area under the curve (AUC) for random forest models constructed with different numbers of KEGG KO functional units.
 (B) ROC curves showing AUC values for random forest models constructed with different numbers of KEGG pathways.
 (C) PCoA of the random forest proximity matrix based on KEGG KO functional units.
 (D) PCoA of the random forest proximity matrix based on KEGG pathways.
 (E) Variable importance ranking of KEGG KO functional units in the random forest model.
 (F) Variable importance ranking of KEGG pathways in the random forest model.
