# Supplementary material for: A metagenomic study of the gut microbiome in patients with type 2 diabetes mellitus and myocardial infarction
Source: Acta Diabetol. 2026 Feb 9;63(5):789–99. doi: 10.1007/s00592-026-02648-x (PMC13219173; doi:10.1007/s00592-026-02648-x)
Supplement: Supplementary file 6 — Supplementary Material 6 [file 592_2026_2648_MOESM6_ESM.docx]

## Conflict of Interest

The authors declare that they have no known competing financial interests or personal relationships that could have appeared to influence the work reported in this paper.
